# Analyses of body composition charts among younger and older Chinese children and adolescents aged 5 to 18 years

**DOI:** 10.1186/1471-2458-12-835

**Published:** 2012-10-01

**Authors:** Kai-Yu Xiong, Hui He, Yi-Ming Zhang, Guo-Xin Ni

**Affiliations:** 1 Teaching experiment center, Beijing Sport University, Beijing, China; 2 Department of Anatomy, College of Sport Science, Beijing Sport University, Beijing, China; 3 Department of Orthopaedics and Traumatology, Nanfang hospital, Southern Medical University, Guangzhou, 510515, China

**Keywords:** Body composition chart, Body mass index, Fat-free mass index, Fat mass index, Chinese children

## Abstract

**Background:**

Childhood obesity has become a major public health problem in China. The objective of this study was to examine the effect of age and sex on the relationship between fat-free mass (FFM) and fat mass (FM), fat-free mass index (FFMI) and fat mass index (FMI) in Chinese children using body composition chart analysis, and to compare the changing pattern with Caucasian and Japanese counterparts.

**Methods:**

A total of 1458 children (790 boys and 668 girls) between 5 and 18 years of age were studied to determine a body composition by bioelectrical impedance analysis. The relationship of FFM and FM, FFMI and FMI were delineated by body composition charts.

**Results:**

Different changing patterns in body composition were observed during 5-11y (younger age group), and 12-18y (older age group), with non-significant sex difference with FM and FMI for the younger age group and significant sex and age differences for the older age group. For the younger age group, simultaneous increase of FFM and FM was found in both genders. However, for the older age group, the increase in weight and BMI with age is largely due to the increment of FFM and FFMI in boys, and of FM and FMI in girls. In addition, different changing patterns in body composition exist between Chinese children and their Caucasian and Japanese counterparts, largely due to the higher fat mass component in Chinese subjects.

**Conclusions:**

Our results indicate that age- and gender-related changing patterns of body composition in Chinese children may differ at different growth stage, and differ with those in Caucasian and Japanese children at the same age period. Such changing patterns should be considered when designing the intervention proposal for childhood obesity in China.

## Background

Obesity has become a major public health problem globally, especially in children [1]. In China, with the recent economic development and lifestyle changes, the prevalence of childhood obesity has increased rapidly [2,3]. National epidemiological survey showed that the prevalence of childhood obesity in 2006 was 8.9% for boys and 5.3% for girls, which was 3.6 and 4.7 times higher than that of 1996 for boys and girls respectively [4]. The ongoing epidemic of childhood obesity has highlighted the importance of growth patterns of body composition. However, little is known regarding the age- and sex-related pattern of changes in body composition in Chinese children [5].

Growth involves the deposition of both fat mass (FM) and fat-free mass (FFM) components. Quantitative information on FM and FFM has been extensively reported on different population [6-9]. However, there is relatively little discussion of the way by which such data is expressed. The body mass index (BMI, weight/height^2^) is commonly used as a simple way of assessing excess weight. Nevertheless, there are well-known limitations regarding the use of BMI as it cannot distinguish FFM and FM, and not sensitive to the change in adiposity during childhood [10-12]. BMI is not able to distinguish whether it is an increase in FM or a decrease in FFM that causes the increase in the body mass of a child, but it can be partitioned into the fat and lean components of FMI (FM index=FM/height^2^) and FFMI (FFM index=FFM/height^2^). It is therefore desirable to investigate the relationship of both compartments (FM and FFM, FMI and FFMI) when analyzing the body composition data.

Currently, there are a number of methods developed to assess the body composition [13]. It is rarely possible to make measurements on large samples with sophisticated techniques, like dual energy X-ray absorptiometry or deuterium dilution. Bioelectrical impedance analysis (BIA) is relatively inexpensive and easy to use, and therefore suitable for epidemiological studies. It can distinguish between lean and fat tissue on the basis of their different conductance and impedance characteristics [14], and has been shown to be reliable and valid, even in children [15-17].

The body composition chart (BCC) was developed to delineate the relationships between FM (FMI) and FFM (FFMI) [18,19]. This simple chart provides four kinds of information at the same time: FM (FMI), FFM (FFMI), body mass index (BMI), and fat percentage, by which those who have a small BMI (slim) without excess fat can be distinguished from the slim who maintain a large FM. Wells [20] used BCC to examine the relationship of BMI and body fatness in reference Caucasian infants and children aged 1–10 years. More recently, Hattori et al. [19] reported the age-related patterns of change in FM/FMI and FFM/FFMI in Japanese subjects aged 11–59 years. Since it is known that human body composition is ethnicity dependent, these body composition charts may not be applicable to children of other ethnicity. This study was therefore carried out to examine the effect of age and sex on the relationship between FM and FFM, and FFMI and FMI in Chinese children in age ranging from 5 to 18 years using body composition chart analysis. A secondary aim was to compare the results of Chinese children with those of Caucasian and Japanese counterparts at the same age period.

## Subjects and methods

### Subjects

A total of 1458 individuals, 790 males and 668 females, between 5 and 18 years of age, living within the urban area of the municipality of Chongqing city, China participated in this study. All participants were recruited from six local primary and secondary schools. Those who are willing to attend this project were selected. They were apparently healthy, and those engaged in any intense sport training programs were excluded. Full informed consent was obtained from the parents/guardians of each child before the start of the study. Ethical permission was granted by the Human Subject Review Committee, Beijing Sport University.

### Measurements

All the measurements were taken in a local fitness club by a single well-trained examiner (H.H.) between October and November, 2011. Subjects were dressed in light sports attire. Height was measured to the nearest 0.01 cm with barefoot, using a stadiometer fixed to a wall in the laboratory. Body weight was measured to the nearest 0.01 kg on an electrical digital scale.

The body composition was assessed with bioelectrical impedance analysis (BIA) using a multi frequency bioelectrical impedance analyzer (InBody J10, Biospace, Seoul, Korea). The measurements were performed according to general recommendations from the manufacturer, at least 2 h after ingestion of a light breakfast and after voiding the urinary bladder before the measurement. The body measurements and BIA were conducted in the morning on the same day.

Two types of body composition charts were used in this study. The details have been described elsewhere [19]. Briefly, the means of FFM and FM for male and female subjects of every age group were plotted on the body composition chart 1 in common units (kg). The x-axis represents FFM and the y-axis FM, with additional diagonal lines indicating weight and fat percentage (% fat). Therefore, four kinds of information were provided at the same time in this chart: FM, FFM, weight, and % fat. The values of body compartment of FM (kg) and FFM (kg) measured with BIA were used to calculate two indices of height-normalized body composition: FFMI, calculated as FFM/height^2^, and FMI, calculated as FM/height^2^. The means of FFMI and FMI for male and female subjects are plotted on body composition chart 2 in common units (kg/m^2^). The x-axis represents FFMI and the y-axis FMI, with additional diagonal lines indicating BMI and % fat. As such, four kinds of information were provided at the same time in this chart: FMI, FFMI, BMI, and % fat.

### Statistical analysis

Based on the age-related changing patterns in both body composition charts, analysis of variance was used to investigate the effect of sex and age on FFM and FM, as well as FFMI and FMI for two growth stages (5-11y, and 12-18y). All statistics were calculated using the SPSS statistical package (version 16.0).

## Results

The means and standard deviations of height, weight, FM, FFM, BMI, FFMI, FMI and fat percentage were presented in Tables 1 and 2.

**Table 1:** Body size and body composition of Chinese boys by age group

**Age group**	**N**	**Height**		**Weight**		**BMI**		**FM**		**FMI**		**FFM**		**FFMI**		**FAT**	
		**Mean**	**SD**	**Mean**	**SD**	**Mean**	**SD**	**Mean**	**SD**	**Mean**	**SD**	**Mean**	**SD**	**Mean**	**SD**	**Mean**	**SD**
5	44	112.82	5.23	22.38	3.79	17.48	1.90	5.66	2.33	4.38	1.59	16.72	1.91	13.10	0.67	24.48	6.41
6	70	118.97	5.20	25.11	4.84	17.67	2.69	6.32	3.36	4.43	2.23	18.79	2.28	13.24	0.92	24.08	8.82
7	105	124.53	5.45	27.73	5.27	17.78	2.50	6.62	3.46	4.21	2.04	21.11	2.60	13.57	0.95	22.73	7.72
8	48	130.61	5.56	30.14	5.16	17.62	2.45	7.08	3.80	4.13	2.07	23.07	2.40	13.49	0.78	22.50	8.07
9	60	133.17	6.51	31.98	6.25	17.94	2.62	7.72	4.05	4.32	2.13	24.26	3.36	13.62	0.92	23.09	7.84
10	49	136.90	6.04	35.37	8.56	18.70	3.44	9.14	5.63	4.77	2.75	26.23	3.76	13.93	1.07	23.98	9.43
11	52	141.75	7.59	37.40	−8.71	18.45	3.04	8.78	5.11	4.32	2.29	28.62	4.99	14.13	1.19	22.31	8.15
12	63	151.85	9.45	47.42	13.61	20.28	4.13	11.35	7.10	4.85	2.83	36.07	8.51	15.42	1.99	22.61	8.69
13	60	158.95	8.27	52.45	10.98	20.64	3.36	10.69	6.44	4.23	2.50	41.76	7.05	16.41	1.57	19.42	8.29
14	50	165.06	5.54	59.52	11.53	21.79	3.85	12.62	7.05	4.62	2.57	46.90	6.50	17.17	1.92	20.10	8.06
15	55	169.92	5.81	63.83	10.54	22.07	3.25	13.63	6.28	4.73	2.20	50.20	6.16	17.34	1.53	20.63	6.78
16	50	169.15	4.56	64.42	8.37	22.48	2.50	12.59	4.92	4.38	1.66	51.83	5.11	18.10	1.47	19.37	5.76
17	39	170.79	9.28	65.13	11.84	22.16	2.92	12.73	6.52	4.31	2.08	52.41	7.55	17.84	1.50	18.83	6.29
18	45	169.07	5.05	67.79	9.60	23.72	3.27	14.49	7.14	5.10	2.60	53.30	4.89	18.62	1.15	20.85	7.69

**Table 2:** Body size and body composition of Chinese girls by age group

**Age group**	**N**	**Height**		**Weight**		**BMI**		**FM**		**FMI**		**FFM**		**FFMI**		**FAT**	
		**Mean**	**SD**	**Mean**	**SD**	**Mean**	**SD**	**Mean**	**SD**	**Mean**	**SD**	**Mean**	**SD**	**Mean**	**SD**	**Mean**	**SD**
5	24	110.76	4.22	20.99	2.62	17.08	1.66	5.28	1.88	4.29	1.45	15.70	1.26	12.82	0.59	24.63	6.17
6	35	116.52	4.94	22.60	4.27	16.56	2.42	5.41	3.07	3.93	2.15	17.19	1.79	12.63	0.63	22.59	8.74
7	44	121.76	6.77	24.86	4.33	16.64	1.76	5.72	2.70	3.81	1.68	19.14	2.45	12.87	0.76	21.96	7.23
8	43	128.19	5.66	28.50	5.87	17.24	2.69	7.25	3.77	4.36	2.12	21.25	2.77	12.88	0.84	24.17	7.78
9	48	133.56	5.75	29.38	4.12	16.43	1.63	6.40	2.39	3.60	1.37	22.99	3.09	12.83	0.91	21.56	6.40
10	35	139.27	7.57	35.07	9.20	17.85	3.23	8.95	4.97	4.51	2.25	26.11	5.06	13.34	1.34	24.08	7.78
11	44	147.48	8.43	41.31	10.37	18.75	3.05	10.71	5.57	4.80	2.16	30.60	5.57	13.95	1.19	24.70	7.21
12	66	152.80	7.81	43.76	8.40	18.61	2.37	10.67	3.92	4.53	1.51	33.09	5.54	14.08	1.28	23.77	5.52
13	52	155.34	10.13	49.75	8.42	20.50	2.23	13.93	4.53	5.76	1.70	35.82	5.45	14.74	1.11	27.64	5.58
14	50	156.72	6.51	50.53	6.87	20.57	2.59	14.84	4.01	6.07	1.76	35.69	4.36	14.50	1.21	29.07	4.86
15	66	158.17	6.07	54.55	7.80	21.78	2.68	17.51	4.79	7.00	1.88	37.04	4.02	14.78	1.05	31.70	4.91
16	61	159.20	5.20	55.19	6.78	21.74	2.11	17.52	4.24	6.91	1.60	37.66	4.02	14.83	1.05	31.58	5.26
17	57	159.14	5.57	54.70	6.35	21.58	2.09	17.60	3.93	6.95	1.51	37.10	3.59	14.63	1.01	31.92	4.44
18	43	157.55	5.04	52.25	4.97	21.06	1.97	15.92	3.58	6.44	1.54	36.33	3.18	14.62	0.90	30.40	5.14

**Figure 1:**
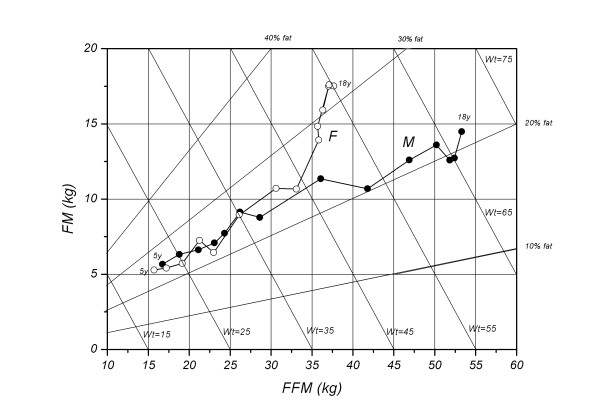
Age-related change of the relation between FM and FFM in Chinese boys and girls aged 5-18y. M: male, F: female.

Figure 1 shows the age-related changes in FM and FFM in male and female subjects from 5 to 18 y old. According to the general changing pattern, two age groups (younger age group: 5-11y, and older age group: 12-18y) were analyzed separately, and the results of analysis of variance showed different changing patterns in body composition with age and sex. Although the FFM showed significant sex and age differences for both groups, the FM for sex difference was non-significant, but was significant for age difference for the younger age group, whereas significant sex and age differences for the older age group (Table 3). Between 5 y and 10 y, the age-related patterns in the boys and girls are similar with simultaneous increase of FFM and FM. During this period, boys exhibit higher FM and FMM than girls at each age point, except 8 y old with higher FM in girls. Age 11 appears to be the only age point where the girls have higher FM and FFM than boys. However, totally different changing patterns were found afterwards, characterized by a sharp rise of FM with a relatively slight increase in FFM for the girls, and a sharp rise of FFM with a relatively slight increase in FM for the boys. In terms of fat percentage, it remains relatively unchanged for both sexes before 12 y, however, it decreases slightly for boys while it increases rapidly for girls afterwards.

**Table 3:** Results of analysis of variance, with age and sex as the factors, of FFMI and FMI of 5–11 y group and 12–18 y group

		**df**	**FFMI**				**FMI**			
			**SS**	**ms**	**F**	**P**	**SS**	**ms**	**F**	**P**
5-11y	Sex	1	43.67	43.67	45.28	<0.01	4.84	4.84	1.06	0.3
	Age group	6	87.19	14.53	15.07	<0.01	40.87	6.81	1.49	0.18
	Sex X age group	6	6.28	1.05	1.09	0.37	25.28	4.21	0.92	0.48
	Residual	687	662.55	0.96			3140.92	4.57		
12-18y	Sex	1	1331.26	1331.26	659.07	<0.01	480.42	480.42	106.96	<0.01
	Age group	6	285.48	47.58	23.56	<0.01	151.71	25.29	5.63	<0.01
	Sex X age group	6	142.81	23.8	11.78	<0.01	184.46	30.74	6.84	<0.01
	Residual	743	1500.78	2.02			3337.25	4.49		

**Figure 2:**
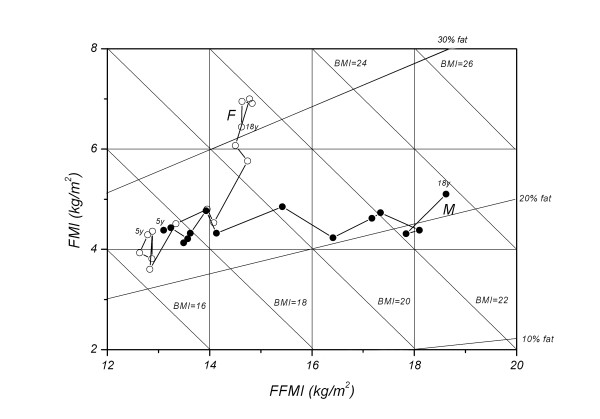
Age-related change of the relation between FMI and FFMI in Chinese boys and girls aged 5-18y. M: male, F: female.

The height-normalized changes in FFM and FM in both male and female subjects are shown in Figure 2. Table 4 showed the results of analysis of variance, with age and sex as the factors, of FFMI and FMI of two age groups. Although the FFMI showed significant sex and age differences for both groups, the FMI for sex difference was non-significant, but was significant for age difference for the younger age group, whereas significant sex and age differences for the older age group. Although boy has higher FFMI at each age point between 5 y and 11 y, plots are dense in a narrow range for both sexes, indicating that the body composition change is proportionate to the change in height squared. In male subjects, the plots from 11 y onwards move directly toward the right-hand side, indicating a consistent increase of FFMI with relatively little change in FMI in the period. In contrast, the plots from 12 y onwards move toward the upper side of the chart for girls, which were characterized by a sharp rise of FMI with relatively little change in FFMI. At each age point, the boys have higher BMI than girls. The increase in male BMI between 5 and 18 y is due to increasing FFMI, while FMI remains relatively unchanged during this period. On the other hand, however, the girls show an increase in both components.

**Table 4:** Results of analysis of variance, with age and sex as the factors, of FFM and FM of 5–11 y group and 12–18 y group

		**df**	**FFM**				**FM**			
			**SS**	**ms**	**F**	**P**	**SS**	**ms**	**F**	**P**
5-11y	Sex	1	104.34	104.34	9.20	<0.01	7.58	7.58	0.463	0.50
	Age group	6	11212.71	1868.79	164.74	<0.01	1429.40	238.23	14.53	<0.01
	Sex X age group	6	275.07	45.85	4.04	<0.01	170.93	28.49	1.74	0.11
	Residual	687	7793.28	11.34			11261.76	16.39		
12-18y	Sex	1	1331.26	1331.26	659.07	<0.01	480.42	480.42	106.96	<0.01
	Age group	6	285.48	47.58	23.56	<0.01	151.71	25.29	5.63	<0.01
	Sex X age group	6	142.81	23.8	11.78	<0.01	184.46	30.74	6.84	<0.01
	Residual	743	1500.78	2.02			3337.25	4.49		

Correlations of BMI with FM, FFM, FMI, and FFMI are shown in Table 5. BMI was positively and significantly correlated with FM and FMI weight for each sex at all ages. Correlations were moderate to high between BMI and FFM and between BMI and FFMI for each sex at each age, with significant difference from zero with the exception to FFM for girls at age 5.

**Table 5:** Correlations of BMI with Body Composition Variables

**Age group**	**Boys**				**Girls**			
	**FM**	**FMI**	**FFM**	**FFMI**	**FM**	**FMI**	**FFM**	**FFMI**
5	0.93*	0.94*	0.59*	0.60*	0.93*	0.95*	0.39	0.62*
6	0.91*	0.93*	0.47*	0.56*	0.97*	0.97*	0.55*	0.60*
7	0.93*	0.93*	0.59*	0.64*	0.92*	0.93*	0.39*	0.51*
8	0.95*	0.96*	0.47*	0.68*	0.96*	0.97*	0.59*	0.76*
9	0.94*	0.95*	0.46*	0.66*	0.90*	0.87*	0.36*	0.55*
10	0.97*	0.96*	0.70*	0.73*	0.95*	0.94*	0.74*	0.83*
11	0.96*	0.95*	0.64*	0.79*	0.97*	0.96*	0.76*	0.86*
12	0.94*	0.90*	0.69*	0.79*	0.90*	0.86*	0.66*	0.81*
13	0.92*	0.90*	0.52*	0.71*	0.87*	0.87*	0.45*	0.66*
14	0.89*	0.90*	0.70*	0.81*	0.91*	0.91*	0.45*	0.81*
15	0.91*	0.92*	0.60*	0.81*	0.94*	0.96*	0.55*	0.86*
16	0.82*	0.79*	0.58*	0.75*	0.87*	0.86*	0.50*	0.63*
17	0.93*	0.92*	0.62*	0.82*	0.84*	0.89*	0.47*	0.74*
18	0.97*	0.96*	0.50*	0.79*	0.90*	0.92*	0.32*	0.71*

* Significantly different from zero *P*<0.05.

**Figure 3:**
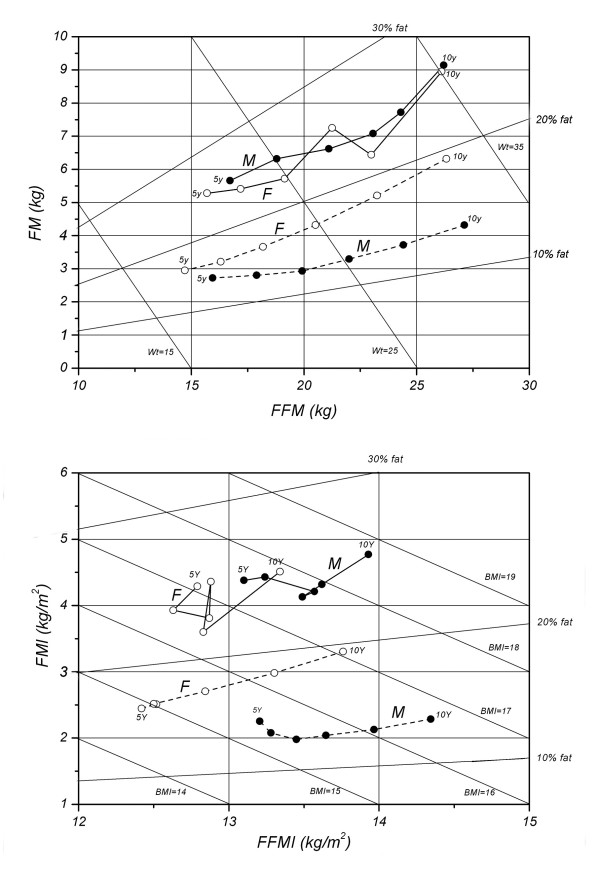
Age-related change of the relation between FM and FFM (UP), FMI and FFMI (BELOW) in Chinese (solid lines) and Caucasian subjects (dashed lines, data from Ref. 21) between 5–10 ys. M: male, F: female.

Figure 3 shows the changing pattern in body composition in Caucasian [20,21] and Chinese subjects between 5 and 10 y. Although both Chinese and Caucasian subjects exhibited simultaneous increase of FFM and FM with age, differences exist in a number of aspects. Caucasian girls had higher weight and BMI than boys, which is opposite to Chinese children. In addition, Chinese boys and girls have considerably higher weight and BMI than their Caucasian counterparts. The fat percentage ranges 20-30% for Chinese children, whereas 10-20% for Caucasian children. Moreover, although Caucasian children had constantly lower FM and FMI at each age point, by 10 y, they had similar FFM and higher FFMI than their Chinese counterparts. 

**Figure 4:**
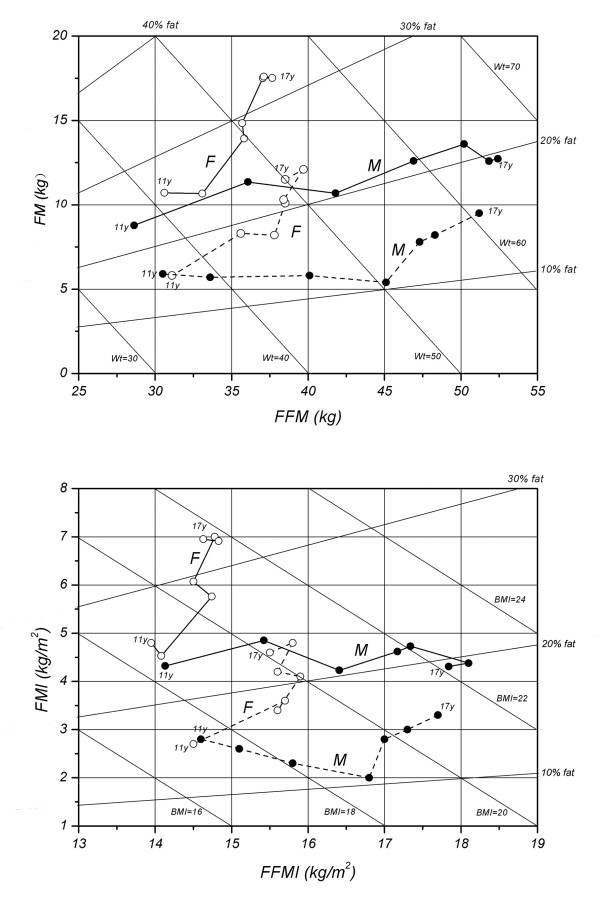
Age-related change of the relation between FM and FFM (LEFT), FMI and FFMI (RIGHT) in Chinese (solid lines) and Japanese subjects (solid lines, data from Ref. 19) between 11–17 ys. M: male, F: female.

Although the general trend in the changing pattern of body composition was similar, great disparity was also observed between Chinese children and their Japanese counterparts [19] between 11-17y (Figure 4). Higher FFM and FFMI were in Japanese girls, however, even higher FM and FMI in Chinese girls led to the higher weight and BMI than their Japanese counterparts. During 11–14 y, Japanese boys had constant FM, and an obvious decline in FMI. However, during the same period, FM increases and FMI remains almost unchanged in Chinese boys. 

## Discussion

In the current study, the relationship between FM and FFM, and FFMI and FMI in Chinese children was, for the first time, examined using body composition chart analysis. These charts graphically present the age and sex-related patterns of changes in body composition of Chinese children aged 5–18 years in this cross-sectional study. Although changes with age cannot be accurately determined from cross-sectional data due to potential cohort or generational differences, the current study indicates several trends in body composition changes during growth period in Chinese children, which are somewhat different from Caucasian and Japanese samples at the same age period.

From 5 to 18 y, the weight and BMI tend to increase with age for Chinese subjects, and Chinese boys tend to have higher weight and BMI than girls. Similar trend has also been reported in a number of studies for Chinese children [22-24]. In Chinese traditional opinion, boys tend to receive extra attention in early life, and are expected to be strong, while girls should be slim. Hence, boys prefer to have a larger body size than girls [25]. What is more, using body composition chart analysis, this current study further illustrates how growth may affect lean and fat development differentially at different times. Between 5 and 11 y, concurrent increase of FFM and FM is generally observed, and the fat percentage was constant for both genders. Therefore, the weight gain is due to both FM and FFM components. The results of analysis of variance, with sex and age group as the factors, revealed that the FM and FMI showed insignificant sex difference, whereas the FFM and FFMI showed significant sex and age differences. As higher FFM is found in boys both on an absolute and height-normalized basis, it is suggested that boys gain relatively more lean mass components than girls during this growing period.

Of interest, totally different changing pattern was found between 12 and 18 y. The results of analysis of variance for this period indicated that FM/FMI and FFM/FFMI showed significant sex and age differences with significant interactions. From 11 to 13 y, fat percentage decreases in boys, but increases in girls. Such gender differences appear to be largely due to the greater production of estrogen by the females and testosterone by the males [26]. The age-related changing patterns are generally characterized by a sharp rise of FM with a relatively slight increase in FFM for girls, and a sharp rise of FFM with a relatively slight increase in FM for boys. Unlike the previous stage, the increase in weight and BMI with age for this stage is largely due to the increment of FFM and FFMI in boys, and of FM and FMI in girls. Therefore, boys’ physiques become more lean and muscular in this growing period. Our findings were in accordance with the report by Chumlea et al. [27], who investigated body composition for children aged 10–18 y, and found that boys have a constant annual increase in lean body mass without a constant annual increase in total body fat; whereas, girls have a small annual change in lean body mass with a constant annual increase in total body fat.

Ethnic differences have been extensively reported on human body composition [28,29]. In the current study, different changing pattern in body composition was also found between Chinese children and their Caucasian and Japanese counterparts using BCC analysis. Between 5 and 10 y, Chinese boys and girls have considerably higher weight and BMI than their Caucasian counterparts, which is almost entirely due to the higher fat mass components in Chinese children. Similarly, the higher weight and BMI in Chinese boys and girls in comparison to their Japanese counterparts between 11-17y is largely due to the higher FM and FMI components. Taken together, our results revealed that the different changing pattern in body composition between Chinese children and their Caucasian and Japanese counterparts largely lies in the higher fat mass component found in Chinese subjects. Such ethnicity-dependent differences may be attributed to a number of factors, including the molecular genetic factor and many other obesogenic environmental factors [29].

With the rapid economical development, the Chinese population, especially those living in urban areas, is undergoing a remarkable but undesirable nutrition, physical and behavioral transition, closely associated with obesity and many other disorders [30-32]. Being aware of this increasing health problem, a number of interventions have been taken for the prevention or treatment of overweight among children and adolescents since the 1990s [33-36]. Nevertheless, the efficacy of these interventions has been mainly inconclusive [37]. As recommended by numerous other studies, our findings also highlight the importance of intervention targeted at the overweight of Chinese children. More importantly, several implications from our findings may be useful for the interventional strategy. Firstly, at early childhood period, Chinese boys tend to have larger body size than girls, which is opposite to that in Caucasian children. Such disparity is very likely due to an indistinct pre-school fatness loss phase [38] for Chinese boys, implying that our intervention should be taken on children, especially boy, as early as possible. Secondly, during early to mid-childhood, the gain in FFMI in Chinese children is much less than Caucasian children. Also, the lean mass components are constantly lower in Chinese girls than Japanese girls from 11 to 17 years. What is more, a typical preadolescent fat loss phase [36], which was observed in Japanese boys between 11 to 14 years, is less evident in Chinese boys. As such, special attention should be paid on the particular component(s), time point(s) and gender(s) when designing the intervention proposal.

There are some limitations to our study. Firstly, our study was carried out in 2011, about 10 years later than Harriot’s [19], and 30 years later than Fomon’s [21]. In addition, BIA was used to assess body composition in our study, whereas, deuterium dilution and densitometry in Fomon’s [21] and Harriot’s [19], respectively. Such inconsistence probably underestimated the differences between Chinese children and their Caucasian and Japanese counterparts. Another concern is that this study was conducted only in Chongqing city. There are regional variations and urban–rural difference in the growth of Chinese children [22,23,31]. Whether or not our findings can be generalizable to children in China needs further investigations on children from different regions of China.

## Conclusion

In conclusion, our results indicate that age- and gender-related changing patterns of body composition in Chinese children may differ at different growth stage. In addition, the changing pattern of body composition in Chinese children is different from that in Caucasian and Japanese counterparts at the same age period, largely due to the higher fat mass component found in Chinese subjects. Our findings have some important clinical implications when designing the intervention proposal for childhood obesity in China.

## Abbreviations

FFM: Fat-free mass; FM: Fat mass; FFMI: Fat-free mass index; FMI: Fat mass index; BMI: Body mass index; BIA: Bioelectrical impedance analysis; BCC: Body composition chart.

## Competing interest

The authors declare no conflict of interest.

## Authors’ contributions

KYX was responsible for the study design. YMZ coordinated the study conduct. HH coordinated the study personnel, the recruitment phase and was responsible for data processing. GXN performed the statistical analysis and prepared the manuscript. All authors read and approved the final manuscript.

## Pre-publication history

The pre-publication history for this paper can be accessed here:

http://www.biomedcentral.com/1471-2458/12/835/prepub
